# The Perfected Treatment of Fibroid Tumors of the Uterus1Presented to the Mississippi Valley Medical Association, at Memphis, Tenn., October 7, 1903. Reprinted from the *Denver Medical Times*, December, 1903.

**Published:** 1904-01

**Authors:** Lewis S. McMurtry

**Affiliations:** Louisville, Ky.; Professor of Gynecology and Abdominal Surgery in the Hospital College of Medicine


### SELECTION.

### The Perfected Treatment of Fibroid Tumors of the Uterus.[1]

By LEWIS S. McMURTRY, M. D., Louisville, Ky.,
Professor of Gynecology and Abdominal Surgery in the Hospital College of Medicine.

THE time allotted each paper on the program does not permit a consideration of the important pathological and clinical considerations appertaining to fibroid tumors of the uterus. It is my purpose to present briefly the results of surgical treatment for uterine fibroids and the methods which have been evolved by many operators to bring about the existing brilliant and satisfactory results of operative treatment.

The large abdominal tumors which so commonly obtain in women are, as a rule, ovarian cysts and uterine fibroids. Both the ovarian and uterine neoplasms mentioned are very common: they destroy life by pressure, hemorrhage, and the degenerative changes to which they are subject, and individuals affected with these growths can only be cured by surgical extirpation of the tumors. Ovariotomy was the first operation established in abdominal surgery, and in connection therewith all the pioneer work was done. Ovarian tumors were from time immemorial recognised as fatal if left to their natural progress, and surgery was invoked for radical cure early in the nineteenth century. Beginning with McDowell's decisive work in 1809, ovariotomy was cultivated by various able and original surgeons in America and Europe until the operation was established and, after Lister's discoveries, quickly advanced to the position of the most successful major operation known to surgery. It was quite different with fibroid tumors of the uterus. With the exception of a few cases, the operation for fibroid tumors of the uterus was not generally considered or practised until a very modern period, and was not placed upon a high plane of diminished mortality until within very recent times. I allude here, of course, to the treatment of interstitial and subperitoneal tumors; the operative treatment of the so-called fibroid polyp, which is an extruded submucous fibroid tumor, being quite simple and outside the peritoneum. As illustrating the recent origin of this great surgical achievement, I may mention that the operative procedure as now practised has been built up by men yet living. At the meeting of this association in Louisville about ten years ago, Dr. R. S. Sutton, of Pittsburg,

---

1. Presented to the Mississippi Valley Medical Association, at Memphis, Tenn., October 7, 1903. Reprinted from the *Denver Medical Times*, December, 1903.

reported a successful operation and predicted that the time would come when the operative treatment of uterine fibroids would equal in results and attain the high position in professional confidence which had obtained then with ovariotomy. That prediction has now been verified, and it is gratifying to record that this great surgical achievement is for the most part the work of American surgeons.

The classical teaching relative to uterine fibroids is in some respects peculiar. Professional authority generally, and even among gynecological writers, was long directed toward establishing a belief in the innocent character of these growths. They were regarded as of minor importance in comparison with ovarian tumors, and it was the classical view that they disappear at the menopause. To urge a suffering woman, breaking down under pressure symptoms and hemorrhage, to be patient until that period, and to administer opium for pain and ergot for hemorrhage was the generally accepted practice. When, in a good proportion of cases, the tumor would undergo degenerative changes, or necrotic or suppurative changes would take place, the growth was pronounced malignant and only mitigating treatment remained to be utilised in the patient's behalf.

Washington L. Atlee, of Philadelphia, a great pioneer in abdominal surgery, portrayed many of the dangers of uterine fibroid tumors in a prize essay which appeared in the Transactions of the American Medical Association in 1853. It was only, however, after Lister's epoch-making discovery that their degenerative possibilities became known and appreciated, a result of the opportunities it afforded to confidently undertake operative treatment and study these tumors in the living. August Martin, in 1890, tabulated the degenerations and complications met with in a series of 205 cases as follows:

| | |
|---|---|
| Fatty degeneration of tumor | 7 |
| Calcification of tumor | 3 |
| Suppuration of tumor | 10 |
| Edema of tumor | 11 |
| Cystic degeneration of tumor | 8 |
| Telangiectasis of tumor | 3 |
| Sarcoma of tumor | 6 |
| Carcinoma of cervix uteri | 2 |
| Carcinoma of corpus uteri | 7 |
| | 57 |

Dr. C. P. Noble, of Philadelphia, in 1903, made observations upon a series of 258 cases, and recorded the following complications:

| | |
|---|---|
| Appendicitis | 9 |
| Bilateral hydrosalpinx | 10 |
| Unilateral hydrosalpinx | 7 |

| | |
|---|---|
| Hematosalpinx | 1 |
| Calcareous infiltration | 5 |
| Cystic degeneration of ovaries | 2 |
| Ovarian cyst with twisted pedicle | 1 |
| Ovarian cyst, bilateral | 2 |
| Ovarian cyst, unilateral | 22 |
| Ovarian cyst, suppurating | 1 |
| Bilateral dermoid cyst, umbilical hernia | 1 |
| Dermoid cyst, suppurating, sinus through abdominal wall | 1 |
| Dermoid cyst with twisted pedicle | 1 |
| Dermoid cyst | 1 |
| Intraligamentous development of fibroid | 18 |
| Retroversion of uterus | 4 |
| Procidentia of uterus | 4 |
| Parovarian cyst | 2 |
| Ectopic pregnancy | 3 |
| Papillary carcinoma of both ovaries | 1 |
| Abscess of ovary | 1 |
| Pyosalpinx, bilateral | 7 |
| Pyosalpinx, unilateral | 4 |
| Salpingitis, bilateral | 2 |
| Salpingitis, unilateral | 8 |
| Myxomatous degeneration of tumor | 8 |
| Cystic degeneration of tumor | 6 |
| Necrosis of tumor | 17 |
| Twisted pedicle, pedunculated tumor | 2 |
| Epitheliomatous infiltration of fibroid tumor | 1 |
| Adenocarcinoma of body of the uterus | 4 |
| Epithelioma of cervix uteri | 4 |
| Sarcoma | 2 |
| Syncytioma | 1 |
| | 163 |

Careful observation has dispelled the classical teaching that fibroid uterine tumors disappear after the menopause. The error was doubtless based on observations of small growths, and on the discontinuance of menstrual excesses. Among the writer's cases are several instances of large tumors which had their most active growth after the menopause. In one notable case a woman at fifty-two was reduced to an extreme condition, bed-ridden and emaciated, with a large uterine fibroid that gave little trouble previous to the menopause. The operation disclosed an immense interstitial fibroid undergoing extensive necrosis in its interior.

The early operations upon fibroid tumors were done in cases where the tumor was mistaken for an ovarian cyst. Burnham, of Lowell, Mass., operated in this way in 1853. Gilman Kimball, of the same town, deliberately operated for a fibroma two months later, imitating McDowell's method of treating the pedicle in ovariotomy. The pedicle (cervix uteri) was transfixed and ligatured with silk, dropped into the pelvis, and the ligatures brought out at the lower angle of the abdominal invasion. This patient recovered, and Dr. Kimball operated eleven times with six recoveries, results now which would scarcely maintain any operation in favor and usage.

Sir Spencer Wells, of England, and the Atlees, in America, while dealing for the most part with ovarian tumors, endeavored to treat uterine fibroids along the same lines. Washington L. Atlee did myomectomy by preference and occasionally did hysteromyomectomy. The operative treatment of uterine fibromata, however, had no established place in surgery during this period, and on account of sepsis and hemorrhage from the pedicle the mortality was too great to secure popular adoption. The first systematic operative procedure was devised and established by Koeberle, of Strassburg, who invented the serre-neude. This eminent surgeon practised the median abdominal incision, and after delivering the tumor incised and stripped away the peritoneum so as to release the bladder in front, and applied the serre-neude to the pedicle; cut the tumor away; fixed the stump in the lower angle of the incision; passed pins above the serre-neude through the stump so as to retain the same in place; brought the peritoneal surfaces together below the neude so as to shut off the peritoneum, and closed the abdominal incision above and around the pedicle. In the skilful hands of Thomas Keith, of Edinburgh, this operation obtained a high position, and after Lister's great discoveries were adopted in surgical practice, this method was followed by Keith in Edinburgh, Tait in Birmingham, Bantock and Thornton in London, Koeberle in Strassburg, and Price in America, with admirable results.

The essential point in hysteromyomectomy from the first related to the treatment of the pedicle. The extraperitoneal treatment of the pedicle with Koeberle's neude was a great advance over all attempts to drop the pedicle after securing it with any kind of ligature. The elastic and contractile tissues of the cervix uteri composing the pedicle were prone to slip from the ligature, and hemorrhage was a common cause of death after operation. The open cervical canal and exposed stump were frequently the infecting focus of septic peritonitis.

Barderheuer instituted the procedure known as pan-hysterectomy, whereby the cervix was completely excised after securing all communicating vessels. This operation was first practised in America in 1888 by Dr. M. A. D. Jones, and has been popularised in France by Doyen, in Germany by August Martin, in England by Christopher Martin, and remains the operation of choice with a number of American operators.

A revolution was wrought in hysteromyomectomy by Dr. L. A. Stimson, of New York, who proposed and practised the ligation of the ovarian and uterine arteries upon each side as the first step in dealing with the tumor. By this simple but essential procedure hemorrhage is controlled without mass ligatures and quickly. From this time the supravaginal amputation with post-peritoneal pedicle increased in favor, and is now the popular established method of doing hysteromyomectomy in the treatment of uterine fibromata. After securing the trunks of the ovarian and uterine arteries on each side, the peritoneum is incised upon the anterior and posterior surfaces of the tumor and stripped away, releasing the bladder in front and making an anterior and posterior flap of peritoneum. The broad ligaments are now divided on each side, the tumor cut away by amputating the cervix low down, the cervix stitched across with absorbable sutures so as to secure small anastomatic vessels, the peritoneal flaps brought together with absorbable sutures across the floor of the pelvis so as to leave the pedicle beneath, thus placing the pedicle outside the peritoneum. The advantages of this procedure consist in ease of execution, brevity of operation, safety of ureters, and the protection afforded against sepsis by complete closure. Indeed the results from this operation are so near perfect as to make the operation equal to that of ovariotomy. The operation is superior to the Koeberle method of extraperitoneal pedicle in that no sloughing stump in the abdominal incision obtains, with the inevitable tendency to ventral hernia at the site of the pedicle. It is superior to pan-hysterectomy in the ease of its execution and the diminished time required.

In 1872, Hegar proposed and practised the removal of the uterine appendages (ovaries and Fallopian tubes) to establish premature menopause and thereby secure atrophy of the fibroid growth. This operation received the sanction of Mr. Lawson Tait, and his great authority secured for it general adoption. It was only a compromise; in some instances on account of the distorted relations of the appendages caused by the growth it was impracticable, and in many instances it failed to secure the desired results. Another procedure practised by some surgeons consisted in ligating the uterine arteries with a view to securing atrophy of the growth, but with unsatisfactory results. The operation already described as supravaginal amputation with extraperitoneal (but intrapelvic) pedicle has yielded such splendid results, that the operation of Hegar and that of ligature of the vessels have been generally discarded.

The operation of myomectomy, by which one or more subperitoneal and interstitial fibroid tumors may be excised and the uterus preserved, has a definite and positive value in the treatment of a certain class of uterine fibromata. In my opinion the efforts of some surgeons toward conservatism in the application of this procedure have gone too far. The great object of every operation is the cure of the patient, maintaining the preservation of all organs and structures compatible with this great purpose. Myomectomy is applicable to subserous tumors whenever single and pedunculated; to multiple sessile and interstitial tumors within certain limits, but judgment should be used in restricting the operation to appropriate cases.

1912 Sixth Street.

